# Epidemiology, clinical characteristics, and outcomes of infective endocarditis over a decade in a tertiary care hospital in Oman

**DOI:** 10.1016/j.ijregi.2026.101033

**Published:** 2026-10-02

**Authors:** Nasma Abdullah Al Sakiti, Ala Al-Lawati, Sachin Jose, Kawther Al Bahrani, Faryal Khamis

**Affiliations:** 1 Department of Infectious Diseases, The Royal Hospital, Muscat, Oman; 2 Department of Cardio-thoracic Surgery, The Royal Hospital, Muscat, Oman; 3 Research Department, Oman Medical Specialty Board, Muscat, Oman; 4 School of Medical Sciences, University of Manchester, Manchester, UK

**Keywords:** Infective endocarditis, Epidemiology, Outcomes, Oman, Diagnosis, *Staphylococcus aureus*

## Abstract

**Objectives:**

Infective endocarditis (IE) remains associated with substantial morbidity and mortality, although data from Oman and the Gulf region are limited. We aimed to describe the epidemiology, clinical characteristics, microbiological profile, and in-hospital outcomes of definite IE in a tertiary care hospital in Oman.

**Methods:**

We conducted a retrospective cohort study of patients admitted between 2006 and 2016. Cases were identified from electronic medical records using the International Classification of Diseases, Tenth Revision, Clinical Modification code I33.0. The modified Duke criteria were applied retrospectively and only definite IE cases were included.

**Results:**

Among 172 screened patients, 130 (75.6%) met the criteria for definite IE. The mean age was 42.3 years, and the median symptom duration before diagnosis was 14 days. Native valve IE occurred in 113 (87.0%) patients and prosthetic valve IE occurred in 17 (13.0%). *Staphylococcus* species were the most commonly isolated pathogens (43.1%), among which *Staphylococcus aureus* accounted for 38.5%. In-hospital mortality was 31.5%, and mortality was lower in patients who underwent surgery than in those who did not (18.0% vs 40.0%; *P* = 0.011).

**Conclusion:**

In this Omani cohort, IE affected a relatively young population and was characterized by predominant *S. aureus* infection and high in-hospital mortality. These findings support context-specific empiric therapy, early echocardiographic evaluation, and timely surgical assessment.

## Highlights

• Decade-long cohort of 130 definite infective endocarditis cases in Oman.
• *Staphylococcus* species caused 43.1% of infections, of which *Staphylococcus aureus* accounted for 38.5%.
• In-hospital mortality was 31.5%; surgery was associated with a lower risk of mortality.
• Only in-hospital outcomes were assessable in this retrospective cohort.
• Findings support early echocardiography and timely surgical assessment.

## Introduction

Infective endocarditis (IE) is defined as an infection of the endocardial surface of the heart, involving one or more heart valves or an intracardiac device. The diagnosis of IE is based on multiple clinical and laboratory parameters; there is no single gold-standard diagnostic tool. Although IE is strongly suspected in patients with predisposing cardiac lesions who present with bacteremia without an obvious source, diagnosing IE in routine practice remains challenging. If the features of IE are atypical or overlap with coexisting diseases, misdiagnosis can be fatal [1]. Conversely, overdiagnosis of IE may lead to numerous iatrogenic issues, including adverse events, overuse of antimicrobial therapy, and complications related to long-term vascular access.

A better understanding of the changing epidemiology of IE can help guide management decisions and treatment planning. Data on the epidemiology and outcomes of IE in the Gulf Region and Middle East remain limited, with only a few published studies available [[2], [3], [4], [5]]. One study from Saudi Arabia described the epidemiologic and clinical features of IE in a cohort of 58 patients and found that native valve IE was the predominant form, with high morbidity and mortality rates [4].

IE in the Middle East warrants dedicated study for several reasons. First, the regional epidemiologic and demographic profile includes a relatively young population, a higher burden of rheumatic heart disease, and a lower prevalence of health care–associated infections than that reported in many Western settings. Second, these factors may give rise to distinct microbiological and clinical patterns that are important for guiding empiric antibiotic selection, timing of surgical referral, and echocardiographic practice. Third, to the best of our knowledge, no large-scale study spanning a full decade had previously been conducted in Oman to characterize these trends [4].

In this study, we analyzed data collected over a decade from a large patient cohort, focusing on the descriptive analysis of IE, which included clinical characteristics, bacteriology, echocardiographic findings, modified Duke criteria scoring, surgical interventions, and patient outcomes.

## Methods

### Study design and setting

We conducted a retrospective cohort study at the Royal Hospital, Muscat, a 1200-bed tertiary care hospital that serves a catchment population of approximately 5.4 million. Patients were identified through the hospital’s electronic medical records using the International Classification of Diseases, Tenth Revision, Clinical Modification (ICD-10-CM) discharge diagnosis code I33.0 for the period from January 2006 to December 2016.

### Study population

The study included adult and pediatric patients. Pediatric patients were defined as those aged 13-17 years and adult patients as those aged ≥18 years. Both groups were analyzed together as a single cohort; where relevant, age-stratified results are presented. Only index hospitalizations were included and patients with multiple admissions were counted once using the first hospitalization that met the diagnostic criteria. The analysis was limited to in-hospital outcomes, and no post-discharge follow-up was available.

### Inclusion and exclusion criteria

Patients were included if they met the modified Duke criteria for definite IE [6], applied retrospectively to available clinical, microbiological, and echocardiographic data. Patients classified as having “possible IE” (n = 42) were excluded because the diagnostic uncertainty in this group would have introduced misclassification bias. Patients aged <13 years were excluded. Cases with insufficient clinical documentation to apply the Duke criteria were also excluded.

### Data collection

The following data were obtained from the electronic medical record: clinical characteristics, including age, sex, comorbidities (diabetes mellitus, hypertension, chronic kidney disease, atherosclerotic valve disease, congenital malformation, valvular heart disease, prosthetic valve, or intracardiac device), intravenous drug use, and the presence of permanent intravascular catheters, including dialysis catheters; laboratory parameters, including white blood cell count, hemoglobin, C-reactive protein, erythrocyte sedimentation rate, and renal and liver function tests; microbiological data, including blood culture results and identified causative microorganisms (*Staphylococcus aureus*, coagulase-negative staphylococci, *Enterococcus faecalis*, Gram-negative organisms, and fungi), as well as cases classified as culture-negative IE; echocardiographic findings assessed by transthoracic echocardiography (TTE) and/or transesophageal echocardiography (TEE), including the side and site of valvular involvement; diagnosis of definite IE according to the modified Duke criteria; antibiotics used, including the most common empirical regimens; the need for surgical valve replacement; and in-hospital mortality.

Symptom duration was defined as the number of days from self-reported symptom onset to the date of hospital admission. Missing data were documented and handled by complete-case analysis; variables with more than 20% missing data are identified in the relevant tables.

### Data analysis

Data were analyzed using IBM SPSS Statistics version 29.0 (IBM Corp., Armonk, NY, USA). Continuous variables are presented using the mean, median, and SD. Categorical variables are presented using frequency and percentage. Associations between two categorical variables were assessed using the chi-square test (Fisher exact test where expected cell counts were <5). A *P* <0.05 was considered statistically significant. The 95% confidence intervals were calculated for key proportions using the Wilson method.

### Ethical considerations

The data used in this study were accessed on August 6, 2017. Ethical approval was granted by the Medical Ethics and Scientific Research Committee (MESRC), Royal Hospital, Muscat, Oman (MESRC 32#/2017). All procedures performed in this study involving human participants were in accordance with the ethical standards of the MESRC, Royal Hospital and with the Declaration of Helsinki. The requirement for informed consent to participate was waived by the MESRC, Royal Hospital because of the retrospective study design and the use of anonymized data. Patient confidentiality was maintained; data were anonymized before analysis. Identifiable records were accessible only to the research team during data collection, in line with institutional protocols.

## Results

A total of 172 patients were identified through ICD-10-CM coding; 130 (75.6%) met the modified Duke criteria for definite IE [6] and were included in the analysis, whereas the remaining 42 (24.4%) were classified as having possible IE and were excluded.

Among patients with definite IE, 66.9% (n = 87) were males and 33.1% (n = 43) were females. The mean age of the patients was 42.3 years, with the largest group (36%) aged 21-45 years, followed by 29% aged 46-60 years, 18% aged 61-75 years, 13.8% aged 13-20 years, and 3% aged 75-99 years, as shown in Table 1.

**Table 1:** Demographics, symptom duration, and echocardiographic findings in patients with definite IE (N = 130).

Characteristic/variable	n (%)
**Sex**	
Male	87 (66.9%)
Female	43 (33.1%)
**Age group (years)**	
13-20	18 (13.8%)
21-45	47 (36.2%)
46-60	38 (29.2%)
61-75	23 (17.7%)
>75	4 (3.1%)
**Duration of symptoms in days**	
≤7	53 (40.8%)
8-14	26 (20.0%)
15-21	10 (7.7%)
22-28	6 (4.6%)
29-35	23 (17.7%)
>35	12 (9.2%)
**Echocardiographic findings, right-sided**	
Tricuspid valve	19 (14.6%)
Pulmonary valve	6 (4.6%)
Both right-sided valves	5 (3.8%)
**Echocardiographic findings, left-sided**	
Mitral valve	57 (43.8%)
Aortic valve	31 (23.8%)
Both left-sided valves	16 (12.3%)

Abbreviation: IE, infective endocarditis.

The most frequent symptoms at presentation were fever (97.7%, n = 127), shortness of breath (72.3%, n = 94), and arthralgia (38.5%, n = 50). The median duration of symptoms before the diagnosis of IE was 14 days (IQR 7-21). The duration of symptoms is shown in Table 1.

Native valve endocarditis occurred in 87% (n = 113) of cases, whereas prosthetic valve endocarditis was observed in 13% (n = 17) [7].

Of all the patients, 30 had right-sided IE and 104 had left-sided IE; four patients had right- and left-sided IE; therefore, these counts are not mutually exclusive. The most frequently involved valves were mitral (43.8%, n = 57) and aortic (23.8%, n = 31), as shown in Table 1.

The valve pathologies and risk factors for endocarditis were as follows: 30.8% of patients had atherosclerotic valve disease and 12.3% had congenital heart disease, with *Streptococcus* species being the most commonly isolated bacteria in these groups. In addition, 9.2% of patients had permanent catheters, mostly dialysis catheters, and 10.8% were people who inject drugs (PWID); in these two groups, the *Staphylococcus* species were the major organisms isolated. One-third (30%) of the patients had no identifiable risk factor.

A total of 41 (31.5%) patients died during the index admission. A total of 50 (38.5%) patients underwent surgical intervention. Of these, 31 (62%) had cardiac valve surgery during the initial admission, whereas 19 (38%) underwent deferred surgery, which was performed more than 3 months after the initial diagnosis. The median time from admission to surgical intervention was 7 days. In the surgical group, 41 of 50 (82%) patients survived, compared with 48 of 80 (60%) of those who did not undergo surgery (*P* = 0.011). Table 2 shows the surgical outcomes in patients with IE who required surgical intervention.

**Table 2:** In-hospital outcomes in patients with definite IE (N = 130).

Part (a) IE-related mortality.			
Characteristic	Alive n (%)	Died n (%)	*P*-value
**Sex**			0.689
Male (n = 87)	61 (70.1%)	26 (29.9%)	
Female (n = 43)	28 (65.1%)	15 (34.9%)	
**Age group (years)**			0.149
13-20 (n = 18)	16 (88.9%)	2 (11.1%)	
21-45 (n = 47)	34 (72.3%)	13 (27.7%)	
46-60 (n = 38)	23 (60.5%)	15 (39.5%)	
61-75 (n = 23)	14 (60.9%)	9 (39.1%)	
>75 (n = 4)	2 (50.0%)	2 (50.0%)	
**Surgical treatment**			0.011^a^
Yes (n = 50)	41 (82.0%)	9 (18.0%)	
No (n = 80)	48 (60.0%)	32 (40.0%)	
**Risk factor**			0.233
No risk factor (n = 39)	27 (69.2%)	12 (30.8%)	
Atherosclerotic valve disease (n = 40)	31 (77.5%)	9 (22.5%)	
Congenital malformation (n = 16)	13 (81.3%)	3 (18.7%)	
Central catheter/dialysis (n = 12)	5 (41.7%)	7 (58.3%)	
PWID (n = 14)	8 (57.1%)	6 (42.9%)	
Pacemaker (n = 2)	1 (50.0%)	1 (50.0%)	
Other (n = 7)	4 (57.1%)	3 (42.9%)	

Part (b) IE-related complications.			
Complication	Percent (%) of patients with complications (n = 79)^a^	Percent (%) of the total cohort (N = 130)	

Heart failure	18 (22.8%)	13.8%	
Embolic stroke	18 (22.8%)	13.8%	
Sepsis	18 (22.8%)	13.8%	
Valvular dysfunction	16 (20.3%)	12.3%	
Pulmonary embolism	6 (7.6%)	4.6%	
Red man syndrome	1 (1.3%)	0.8%	
Cardiac tamponade	1 (1.3%)	0.8%	
Lost to follow-up	1 (1.3%)	0.8%	

a Overall complications: 79 of 130 (60.8%; 95% confidence interval 51.8-69.2%). Percentages below are shown as a proportion of the 79 patients who developed complications and as a proportion of the total cohort of 130 patients. Abbreviations: IE, infective endocarditis; PWID, people who inject drugs.

The reasons for surgical intervention were severe valvular dysfunction in 30 (60%) patients; vegetation size for the prevention of embolic events in nine (18%) patients; and uncontrolled infection, including perivalvular abscess, prosthetic valve endocarditis, and other causes, in 11 (22%) patients. Among the 41 patients who died, the interval between the initial diagnosis of IE and death ranged from approximately 22 to 30 days (approximately 3-4 weeks).

Of the 130 patients, 79 (60.8%) developed at least one complication. Among the patients who developed complications, heart failure, embolic stroke, and sepsis each occurred in 18 patients (22.8%); valvular dysfunction occurred in 16 (20.3%); pulmonary embolism occurred in six (7.6%); and red man syndrome, cardiac tamponade, and loss to follow-up occurred in one (1.3%) patient each. Table 2 illustrates the IE-related complications.

The most common organisms observed in patients with IE were staphylococci, accounting for 56 (43.1%) of all the cases, among which *S. aureus* was isolated in 50 (38.5%) of the patients, whereas 39 (30.0%) had methicillin-sensitive *S. aureus* (MSSA), 11 (8.5%) had methicillin-resistant *S. aureus* (MRSA), and six (4.6%) had coagulase-negative staphylococci. The second most common organisms causing IE were *Streptococcus* species, isolated in 38 (29.2%) patients, followed by culture-negative IE in 21 (16.2%), Gram-negative organisms in 12 (9.2%), *E. faecalis* in eight (6.2%), and fungal IE in 1 (0.8%). Table 3 summarizes the distribution of isolated pathogens by risk factors.

**Table 3:** Distribution of isolated pathogens stratified by primary risk factor in patients with definite IE (N = 130).

Risk factor	Total n (%)	MSSA n (%)	MRSA n (%)	CoNS^a^ n (%)	*Strep.* spp n (%)	*E. faecalis* n (%)	Gram-negative organism n (%)	Fungal organisms n (%)	Culture-negative n (%)
No risk factor	39 (30.0%)	10 (25.6%)	2 (5.1%)	—	11 (28.2%)	3 (7.7%)	4 (10.3%)	0	9 (23.1%)
Atherosclerotic valve disease	40 (30.8%)	8 (20.0%)	2 (5.0%)	—	15 (37.5%)	3 (7.5%)	4 (10.0%)	1 (2.5%)	7 (17.5%)
Congenital malformation	16 (12.3%)	3 (18.8%)	0	—	9 (56.3%)	0	2 (12.5%)	0	2 (12.5%)
Central catheter/dialysis	12 (9.2%)	6 (50.0%)	2 (16.7%)	—	0	0	1 (8.3%)	0	3 (25.0%)
PWID	14 (10.8%)	9 (64.3%)	4 (28.6%)	—	1 (7.1%)	0	0	0	0
Pacemaker	2 (1.5%)	1 (50.0%)	0	—	1 (50.0%)	0	0	0	0
Other	7 (5.4%)	2 (28.6%)	1 (14.3%)	—	1 (14.3%)	2 (28.6%)	1 (14.3%)	0	0
Total	130 (100%)	39 (30.0%)	11 (8.5%)	6 (4.6%)	38 (29.2%)	8 (6.2%)	12 (9.2%)	1 (0.8%)	21 (16.2%)

a Risk factor–stratified CoNS counts were not consistently available in the retrospective data set and are, therefore, shown only in the total row. Abbreviations: CoNS*,* coagulase-negative staphylococci; *E. faecalis, Enterococcus faecalis*; MSSA, methicillin-sensitive *Staphylococcus aureus*; MRSA, methicillin-resistant *Staphylococcus aureus*; PWID, people who inject drugs; *Strep.* spp*, Streptococcus* species*.*

The most frequently used antimicrobial agents in conservative medical management were vancomycin, benzylpenicillin, gentamicin, ceftriaxone, and ampicillin, selected according to blood culture results and antimicrobial susceptibility testing and administered parenterally for approximately 6 weeks.

PWID were more commonly infected with MSSA (64.3%) and MRSA (28.6%) than with other pathogens. Patients with catheters or undergoing dialysis showed higher rates of MSSA (50.0%) and MRSA (16.7%). Patients with atherosclerosis most frequently had *Streptococcus* species (37.5%) and *E. faecalis* (7.5%). Congenital malformations were associated with *Streptococcus* species in 56.3% of cases. Table 3 shows the distribution of various risk factors across different pathogens isolated from patients diagnosed with IE.

## Discussion

This retrospective study describes the epidemiology, clinical characteristics, time to diagnosis, and outcomes of patients with IE admitted to a tertiary center in Oman over a 10-year period.

In this study, 65.4% of patients were in the young (21-45 years) and middle-aged (46-60 years) groups. This is consistent with the findings of Al Abri *et al*. who reported a median age of 43.6 years [4]. In contrast, the ICE Prospective Cohort Study reported a mean age of 56.5 years across 58 hospitals in 25 countries [8]. Similarly, a United States–based study reported a mean age of 57 years, which is substantially older than that of our cohort. Studies from Saudi Arabia have reported variable mean ages, ranging from 32 to 59.7 years in different cohorts [2,3]. More recent studies have also described a shift in IE demographics, with increasing numbers of cases occurring in older patients and in those with health care–associated infections [9,10]. The variation in mean age across studies likely reflects differences in the populations affected, as well as local demographic characteristics and risk factor profiles.

Most (60.8%) patients in this cohort presented within 2 weeks of symptom onset [11]. The ICE study reported a median symptom duration of approximately 21 days [8]. The relatively short interval between symptom onset and diagnosis in our cohort may reflect the fact that many patients presented with active bacteremia and underlying cardiac disease. More indolent presentations of endocarditis are typically associated with foreign material, such as prosthetic valves or pacemakers, and with streptococcal infection.

When IE is clinically suspected, blood cultures should be taken and echocardiography performed without delay [[12], [13], [14], [15]]. In this cohort, echocardiography showed that the mitral valve was the most commonly affected valve, followed by the aortic valve. These findings are similar to those from two studies from Saudi Arabia [2,3]. More recent data show that the aortic valve has overtaken the mitral valve as the most commonly affected site [9,16]. This shift likely reflects changes in patient demographics, greater use of prosthetic valves, and evolving microbial patterns. The mitral valve predominance seen in this study fits with the younger age profile and the higher rates of rheumatic heart disease in the Omani population.

The most frequent bacteria isolated were *Staphylococcus* and *Streptococcus* species, consistent with regional and international studies [2,3,8]. *S. aureus* accounted for 38.5% of the isolates as the single most common pathogen, whereas *Staphylococcus* spp. accounted for 43.1% of the cases. Most (70%) patients with IE had an identifiable underlying predisposing condition. The most common predisposing condition was atherosclerotic valve disease, followed by congenital heart defects. *Streptococcus* species were more prevalent in those with structural heart disease, whereas the *Staphylococcus* species predominated in patients with central catheters and PWID. These patterns follow those reported in regional and international series and contribute to the limited literature on IE in this region [[3], [4], [5]].

Overall, 38.5% of patients underwent valvular surgical intervention. The mortality was 18% in the surgical group compared with 40% in those who did not undergo surgery; this difference was statistically significant. Our findings are consistent with current guidelines, which recommend early surgical review when IE is complicated by heart failure, uncontrolled infection, or high embolic risk [12,13,17]. This observation is also in keeping with a 2024 meta-analysis that found a significant advantage for surgery over medical management alone [18]. The mortality rate in our surgical group was lower than that reported in some published series, where surgical mortality has been as high as 50% [13,19,20].

The median time from admission to surgical intervention in our cohort was 7 days. The 2023 European Society of Cardiology (ESC) guidelines now recommend a more stratified approach to surgical urgency, classifying cases as emergency, urgent, or non-urgent based on clinical presentation [21,22]. Our cohort’s median time of 7 days fits the urgent category under previous guidelines, although it sits at the boundary of the more recently tightened threshold. For patients who developed stroke, the optimal surgical timing requires careful consideration; early surgery within 7 days of ischemic stroke was not associated with excess mortality in one study [23], whereas a meta-analysis of 22 studies found higher peri-operative mortality when surgery was performed within 14 days of stroke [24]. The 2023 ESC Guidelines recommend that ischemic stroke alone should not delay surgery when a clear cardiac indication exists, whereas hemorrhagic stroke warrants deferral for up to 4 weeks [21,22]. These observations support the role of a formal multidisciplinary endocarditis team in guiding surgical timing decisions and ensuring adherence to current guidelines [25].

In this study, stroke was among the most common IE-related complications, occurring in 22.8% of patients who developed complications, alongside heart failure and sepsis (each occurring in 22.8%). Stroke in IE was assumed to result from emboli originating from vegetations on infected valves. Vegetations larger than 10 mm have been particularly linked to a higher risk of embolization [26,27]. Delayed recognition in patients with non-specific or overlapping clinical features may allow subacute forms of the disease to progress insidiously, leading to larger vegetations and a higher risk of embolic events, whereas *S. aureus* infection more often follows an abrupt course with pronounced manifestations.

The in-hospital mortality rate of 31.5% in this cohort is higher than rates reported in recent large international studies, including 14.5% in a Japanese nationwide database study [28] and 19.9% in a Swiss cohort [29]. This difference may reflect delayed presentation, limited access to advanced diagnostic methods, including TEE and cross-sectional imaging, during the study period, and the high prevalence of *S. aureus* IE in our cohort.

## Limitations

This study has a number of limitations. Being a retrospective analysis from a single tertiary center, the results may not fully reflect the broader population or other health care settings. In addition, some clinical data were incomplete or missing, which may have introduced bias in patient selection or outcome assessment; for example, the exact proportions of transthoracic vs TEE; vegetation size; use of advanced imaging modalities (brain magnetic resonance imaging or whole-body computed tomography); and extracardiac embolic or metastatic infectious complications, such as splenic emboli, renal emboli, spondylodiscitis, and osteitis, were not systematically documented. Moreover, apart from in-hospital mortality, the lack of long-term follow-up limited our ability to evaluate outcomes beyond the index admission. Furthermore, the absence of a multivariate analysis means that the association between surgical intervention and survival should be interpreted with caution, given the potential confounding.

## Conclusions

This study adds to our understanding of the epidemiology, clinical features, microbiological patterns, and outcomes of IE in Oman. The predominance of *S. aureus* and *Streptococcus* species indicates the need for prompt microbiological investigation and appropriately targeted empiric therapy. The data suggest that early surgical cardiac intervention may improve outcomes. Future multicenter prospective studies would help validate these findings and support efforts to improve early diagnosis, treatment planning, and long-term follow-up for patients with IE in the Gulf Region.

## Funding

This research did not receive any specific grant from funding agencies in the public, commercial, or not-for-profit sectors.

## Ethical approval

This study was approved by the MESRC, Royal Hospital, Muscat, Oman (MESRC 32#/2017). All procedures performed in this study involving human participants were in accordance with the ethical standards of the MESRC, Royal Hospital and with the Declaration of Helsinki.

## Data availability

The data sets generated and/or analyzed during the current study are not publicly available due to patient privacy and institutional restrictions but are available from the corresponding author on reasonable request, subject to approval by the relevant institutional authority.

## Consent to participate

The requirement for informed consent to participate was waived by the MESRC, Royal Hospital, Muscat, Oman because of the retrospective study design and the use of anonymized data.

## Consent for publication

Not applicable.

## Artificial intelligence use disclosure

During the preparation of this work, the authors used Genspark AI Workspace 4.0 to improve the language, readability, and overall clarity of the manuscript. After using this tool/service, the authors reviewed and edited the content as needed and take full responsibility for the content of the published article.

## CRediT authorship contribution statement

**Nasma Abdullah Al Sakiti:** Formal analysis, Writing – original draft, Writing – review & editing. **Ala Al-Lawati:** Data curation, Supervision, Writing – review & editing. **Sachin Jose:** Data curation, Formal analysis, Investigation, Methodology, Software, Validation. **Kawther Al Bahrani:** Data curation. **Faryal Khamis:** Conceptualization, Methodology, Project administration, Supervision, Writing – review & editing.

## Declaration of competing interest

The authors have no competing interests to declare.
